# Complete Genome Sequence of Polychlorinated Biphenyl Degrader *Comamonas testosteroni* TK102 (NBRC 109938)

**DOI:** 10.1128/genomeA.00865-14

**Published:** 2014-09-11

**Authors:** Kohei Fukuda, Akira Hosoyama, Keiko Tsuchikane, Shoko Ohji, Atsushi Yamazoe, Nobuyuki Fujita, Masaki Shintani, Kazuhide Kimbara

**Affiliations:** a Department of Bioscience, Graduate School of Science and Technology, Shizuoka University, Naka-ku, Hamamatsu, Japan; b Biological Resource Center, National Institute of Technology and Evaluation, Nishihara, Shibuya-ku, Tokyo, Japan; c Department of Applied Chemistry and Biochemical Engineering, Graduate School of Engineering, Shizuoka University, Naka-ku, Hamamatsu, Japan

## Abstract

*Comamonas testosteroni* TK102 (NBRC 109938; JCM 19603) can utilize biphenyl as a sole carbon source and degrade polychlorinated biphenyls (PCBs). The complete nucleotide sequence of the TK102 genome was determined. TK102 possesses several integrative and conjugative element-like regions, and one of them carries biphenyl-degradative genes.

## GENOME ANNOUNCEMENT

*Comamonas testosteroni* TK102 (NBRC 109938; JCM 19603), isolated from soil contaminated with polychlorinated biphenyls (PCBs) in Hamamatsu, Japan, can grow on biphenyl and degrade PCBs by a co-metabolism process (1). The biphenyl-degradative operon, *bphA1A2A3BCD*, for the upper pathway to transform biphenyl to benzoate was previously identified in TK102 (2). However, a large number of the genes for the lower biphenyl pathway in TK102 were unknown. To elucidate the responsible genes for biphenyl metabolism, we decided to determine the genome sequence of TK102.

The genome of TK102 was determined using a combined strategy of GS FLX Titanium, HiSeq 1000 (Illumina), and MiSeq technologies. A total of 356,679,340 base sequences (58.8-fold genome coverage; 636,417 reads) from the GS FLX Titanium system, 374,799,567 base sequences (61.8-fold genome coverage; 3,778,312 reads) from the HiSeq 1000, and 90,856,270 base sequences (15.0-fold genome coverage; 1,795,467 reads) from the MiSeq were used for assembly. The reads were assembled with Newbler v2.6 software (3) to yield 150 contigs in 26 scaffolds. The remaining gaps between the scaffolds were closed by additional sequencing of PCR products and fosmid clones using an ABI 3730 sequencer. Sequence annotation was performed using the NCBI Prokaryotic Genome Automatic Annotation Pipeline (http://www.ncbi.nlm.nih.gov/genome/annotation_prok/), and the resulting annotation was manually inspected with respect to the start codon positions using the Microbial Genome Annotation Pipeline (http://www.migap.org/) as well as another annotation support tool of GenomeMatcher (4).

The TK102 chromosome is 6,062,703-bp (62% G+C content), and has 5,544 coding sequences (CDSs), 10 sets of rRNA genes, and 88 tRNA genes. Benzoate is an intermediate compound of biphenyl metabolism in TK102. The benzoate metabolism via catechol is well known as the Ben-Cat pathway (5), but genes for catechol-1,2-dioxygenase (*catA*) were not found in the TK102 genome. Putative genes encoding meta-cleavage enzymes specific for catechol were not found either. On the other hand, putative genes for the Box pathway, in which the benzoate is metabolized via benzoyl-coenzyme A (6), were found. These genes were tentatively named *boxABC* and *adh*. Notably, 6 DNA regions were found, which were predicted to be integrative and conjugative elements (ICEs). The *bph* genes involved in PCB degradation are located on one of the ICE-like regions. The nucleotide sequences of the region showed 97% identity with those of ICE _KKS102_4677 found in another PCB-degrader, *Acidovorax* sp. KKS102 (7). While the 16S rRNA gene of TK102 showed the highest similarity (99.9%) with that of *C. testosteroni* NBRC 14951^T^, the average nucleotide identity based on BLAST (ANIb) between strains TK102 and NBRC 14951^T^ (DDBJ/EMBL/GenBank accession no. BBJZ01000001 to BBJZ01000065) was 92.3%, showing a lower value than the 95% to 96% threshold used to distinguish between bacterial species (8). This result suggests that TK102 might be taxonomically different from *C. testosteroni.*

### Nucleotide sequence accession number.

The nucleotide sequences of the TK102 chromosome were deposited at DDBJ/EMBL/GenBank under accession no. CP006704.
